# Sustainable entrepreneurship out of entrepreneurial opportunity identification: The mediating role of psychological capital

**DOI:** 10.3389/fpsyg.2023.1129855

**Published:** 2023-03-28

**Authors:** Hongxin Zhang, Hongxia Chen

**Affiliations:** ^1^Institute for Zhongyuan Peasant Studies, Zhoukou Normal University, Zhoukou, China; ^2^School of Marxism, Zhoukou Normal University, Zhoukou, Henan, China

**Keywords:** entrepreneurial intention, entrepreneurial opportunity identification, psychological capital, university students, entrepreneurship education

## Abstract

**Introduction:**

The aim of the present study, was to examine the simultaneous effects of entrepreneurial opportunity identification (EOI) and psychological capital (PC) on university students' entrepreneurial intention (EI). Compared with necessity-driven entrepreneurship, opportunity-driven entrepreneurship is more sustainable. Scholars have shown that EOI is key to forming EI, but little has been discussed about its association with PC.

**Methods:**

A total of 555 university students in China were enrolled by means of convenience sampling. Descriptive statistics and correlation analysis of variables were performed using SPSS 21.0 software. Structural equation modeling analysis (SEM) with AMOS 21.0 was used to examine the structural effects of EOI and PC on university students' EI.

**Results:**

According to the results, university students' EOI and PC had a positive and insignificant influence on their levels of EI. Furthermore, PC was found to fully mediate the impact of EOI on EI.

**Discussion:**

The present study could shed light on new instructions to examine the interaction between the cognitive and psychological components of EI in the field of entrepreneurship. It is recommended that educators and practitioners should pay regard to the role of EOI and PC.

## Introduction

As a main driver of socioeconomic growth, innovation, employment, and tackling poverty (Shane and Venkataraman, 2000; Abdelwahed, 2022; Zhang et al., 2022), theoretically and practically, entrepreneurship has become an important issue to be dealt with. Previous studies highlighted that intentions are seen as a key factor in predicting potential entrepreneurs' decisions (Schlaegel and Koenig, 2013; Donaldson, 2019; Antončič and Auer Antončič, 2023; Yang et al., 2023). On the possibility of shaping entrepreneurial intention (EI), university students are considered the most potential groups; thus, it is not surprising that governments from all over the world now have been offering entrepreneurial education courses to increase university students' EI (Pandit et al., 2018; Al-Harasi et al., 2021; Luo et al., 2022; Soomro and Shah, 2022; Wang et al., 2022). The fact is that, however, compared with other career options, only a few of these students engage in entrepreneurial activities and become entrepreneurs (Bae et al., 2014; Galvao et al., 2018; Bazkiaei et al., 2020). Thus, there is still limited comprehension of how entrepreneurial education influences EI among university students.

As many previous studies stated, one of the immediate outcomes resulting from entrepreneurship education is expected to enhance students with the skills to identify business opportunities (Liñán et al., 2011; Hou et al., 2022). In this vein, some scholars had viewed opportunity identification as an antecedent for predicting EI in the field of business (Bao et al., 2017; Mahmood et al., 2019; Wang et al., 2019). Indeed, compared with necessity-driven entrepreneurship, opportunity-driven entrepreneurship is more sustainable (Patzelt and Shepherd, 2010; Karimi et al., 2017). Logically, however, EOI alone is not sufficient to predict EI. Other antecedents, such as positive psychological characteristics, also play an essential role in developing EI. For example, a study conducted by Karimi et al. (2017) among 452 agriculture undergraduate students in Iran and Afghanistan found that psychological motivations motivate students' EI more than economic motivations. Among psychological characteristics, PC is a strong predictor of EI and it is positively associated with successful entrepreneurship (Contreras et al., 2017; Choi and Hwang, 2020). The contribution of PC in explaining EI is obvious since previous research in the field of business shows that entrepreneurs differ from no entrepreneurs in terms of essential PC ingredients (Bockorny and Youssef-Morgan, 2019; Chen and Tao, 2021). For those who would be entrepreneurs, the positive effect of self-efficacy on EI is well-documented (e.g., Gao and Qin, 2022; Niu et al., 2022).

Moreover, although the separate effects of EOI and PC on EI have been widely known and empirically tested in the field of business, however, the empirical literature about how it can be applied to university students' EI is still limited and unknown. To the best of our knowledge, no studies have examined the simultaneous effects of EOI and PC on EI among university students. It is also unclear whether and to what extent PC mediates the relationship between EOI and EI. Therefore, to address this research gap, in a sample of Chinese university students, the present study aimed to examine the role of entrepreneurial opportunity identification (EOI) in shaping the EI of university students. In particular, the mediating role of PC between EOI and EI on university students' EI was tested.

The present study is organized into six separate sections. The next section showed a literature review and hypotheses development to propose a model of how EOI and PC predict EI. This is followed by the Materials and methodology section including the details of the participants, the measurement of the scales and their model-data fit, and the method of data analysis. Thereafter, the results of the present study were conducted. In the next section, we presented a detailed discussion, including the main findings, theoretical contributions and practical implications, and limitations and future research. Finally, the conclusion was presented.

## Literature review and hypothesis development

### Entrepreneurial opportunity identification and entrepreneurial intention

The creation of a new venture is practiced not so much in words as in attitude and intentions. In the entrepreneurship decision process, one of the cognitive factors considered by an individual is entrepreneurial opportunity identification (Kirzner, 1979; Krueger, 2007; Ozgen and Baron, 2007; Shepherd et al., 2015; Lin et al., 2021; Hoang et al., 2022). Opportunity identification is a cognitive process by which ideas for possible business ventures are identified by an individual. In practice, an entrepreneur can identify chances based on various sources of information (Zahra et al., 2009; Hills et al., 2011). In the field of business, scholars have agreed that a potential entrepreneur's effort to create a new venture is triggered by perceptions of opportunity (Grégoire et al., 2010; Song et al., 2017; Shu et al., 2018; Sakib et al., 2022). Those who perceive a business opportunity to be desirable and feasible are more likely to show a greater inclination toward a new venture start-up. For example, a study conducted by Mahmood et al. (2019), in a sample of 310 Asnaf millennials, found that resource and opportunity recognition had a statistically significant effect on pre-startup behavior through EI.

In the field of education, as potential entrepreneurs, university students' ability of EOI is also a crucial area of concern. Recently, some studies have posited a positive association between EOI and EI among university students. For instance, in a study of 466 Chinese university students, Wang et al. (2019) findings show that university students' sense of opportunity identification efficacy can significantly and positively stimulate their social EI, and the network embeddedness is also correlated with their sense of opportunity identification efficacy. Therefore, EOI may have a constructive effect on EI. By focusing on the same aspects, using data from 334 Indian university students, Hassan et al. (2020) concluded that self-efficacy opportunity recognition also shows a significant positive impact on the EI of students, and gender negatively moderates “opportunity recognition–intention” and “self-efficacy–intention” relationships. Similarly, Hou et al. (2022) also empirically confirmed that entrepreneurship education can promote the EI of students through opportunity recognition in a sample of 1,150 university students in China. More recently, a study conducted by Abdelwahed (2022), in a sample of 292 Saudi Arabia's university students, found a positive and significant effect of attitudes toward sustainability, perceived desirability, and perceived feasibility on sustainable EI and opportunity recognition; the opportunity identification factor also significantly and positively affects sustainable EI.

Hence, the present study proposes the following first hypothesis:

*H1. EOI positively and significantly affects EI among university students*.

### Psychological capital and EI

According to Luthans et al. (2007a), PC is related to individuals' positive progress, consisting of self-sufficiency, optimism, hope, and resilience. Originally, as a psychological construct in the field of organizational management, previous studies have concentrated mainly on the positive relationship between PC and employees' engagement, employees' innovative intention, job satisfaction, business excellence, and organizational performance (Luthans et al., 2007b; Nolzen, 2018; Alshebami, 2021; Saleem et al., 2022; Zhang et al., 2022). Entrepreneurs' mental states and entrepreneurial ideas play a key role in their entrepreneurial decisions; hence, the effects of PC on entrepreneurial activity may also exist in the field of business (e.g., Baluku et al., 2016; Su et al., 2020; Xie et al., 2022).

In education, studies have demonstrated that several dimensions of PC, i.e., self-efficacious (help students to have faith in their skills and insights), optimistic (help students to identify business opportunities where others see disorder), hope (help students to focus on different ways to attain their goals), and resilient (help students to bounce back from failures and adversity), both theoretically and empirically, could influence university students' EIs and entrepreneurial success and are a vital initiator (Wu et al., 2019; Wang and Huang, 2022).

Recently, it is worth noting that the synergy effect of PC on entrepreneurship in the field of education is emerging. For example, using a sample of 384 entrepreneurs in Uganda, Baluku et al. (2016) observed that both startup capital and PC are significant predictors of EI and entrepreneurial success; compared with startup capital, PC is the better predictor. Moreover, using a total of 1,914 university students in China, Zhao et al. (2020) showed that PC has a significant indirect impact on students' EI through traditional, financial, human, and social capital. Similarly, in a sample of 564 university students in Northern Cyprus, Maslakci et al. (2021) found that both improving university students' attitudes toward multiculturalism and enhancing their PC will have a beneficial effect on their EI.

Based on the aforementioned studies, the present study proposes the second hypothesis:

*H2. University students' PC is positively and significantly associated with their higher levels of* EI.

### PC as a mediator

Psychological capital not only influences EI but also could directly affect the ability of entrepreneurs to acquire financial, human, and social capital. Since EOI helps entrepreneurs grasp the changing markets and make timely adjustments, thus generating positive feedback on their entrepreneurial psychology (Dheer and Lenartowicz, 2018; Ndofirepi, 2020). Individuals with a higher sense of self-sufficiency are more likely to be secure and have a greater ability to cope with challenges. Thus, between university students' EOI and their EI, PC may have a mediating role.

On the one hand, according to EEM (Shapero and Sokol, 1982), two antecedents, namely perceived desirability and perceived feasibility (such as enabling factors, emotional, social, and cognitive competencies), are of importance in individuals' entrepreneurial activities. The increased sense and abilities of university students from EOI would strengthen their perceived feasibility toward entrepreneurship, encourage them to pursue their goals, and advance their hopes. Moreover, university students' increased PC should have a positive effect on their entrepreneurship attitudes, increase their hopes for the future, and improve their sense of self-sufficiency by enhancing their ability to cope with difficult situations.

On the other hand, according to the assumption of the theory of planned behavior (TPB) (Ajzen, 1991; Fishbein and Ajzen, 2011), previous studies have indicated that psychological characteristics and cognitive variables, such as the propensity to risk, the need for achievement, and cognitions, could mediate the relationship between students' personal factors and EI (Digan et al., 2019; Wu et al., 2019; Ndofirepi, 2020; Tan et al., 2021; Guo et al., 2022). For example, a recent study conducted by Mahfud et al. (2020) noted that the PC as the mediator could influence students' entrepreneurial attitude toward EI to start new businesses. Furthermore, Maslakci et al. (2021) have also indicated that PC is a mediator variable in the relationship between multicultural attitudes and EI. More recently, using a sample of 380 Chinese students, Na et al. (2022) findings show that students' delayed contentment significantly and positively affects their levels of EI, and PC mediates this process.

Based on the aforementioned arguments, the present study proposes the third hypothesis as follows:

*H3. PC mediates the relationship between university students' EOI and EI*.

### Rationale for the present study

As per the entrepreneurial event model (EEM) (Shapero and Sokol, 1982), three antecedents, namely perceived desirability, perceived feasibility, and propensity to act, play a decisive part in predicting EI. Previous studies indicated that individuals with high levels of sense and ability of EOI could perceive high feasibility, which, in turn, enhance their EIs (Corner and Ho, 2010; Hanohov and Baldacchino, 2017). On the other hand, a positive relationship between PC and students' EI could be warranted by the social cognitive career theory (Lent et al., 1993; Sheu et al., 2010), which links career decisions to the perceptions of self-sufficiency and result in expectations. From the positive psychology, considering the synergy effect of PC attributes may be greater than the sum of sub-dimensions (Luthans et al., 2007a), it is reasonable to posit that the interactive effects of EOI, PC, and EI should exist.

**Figure 1:**
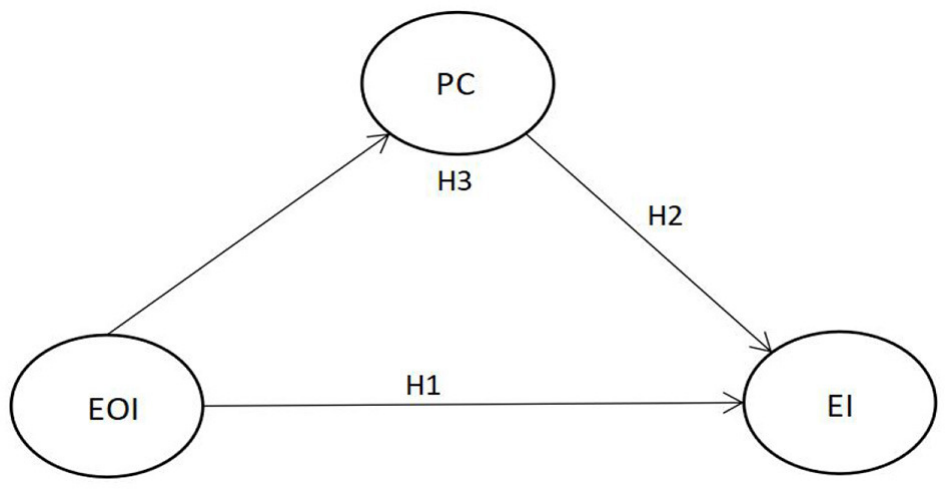
Proposed mediation model. EOI, entrepreneurial opportunity identification; PC, psychological capital; EI, entrepreneurial intention.

Taken together, based on the aforementioned analysis and previous studies, the present study's conceptual framework is shown in Figure 1.

## Materials and methods

### Participants

The Ethics Committee of the Zhoukou Normal University approved the present study. Participants were enrolled by means of convenience sampling at a single university from a university in Zhoukou of Henan Province, China. An online questionnaire was designed since no questionnaire was submitted until all items were completed, and there was no incomplete questionnaire. An online questionnaire survey was distributed with the platform Questionnaire Star assisted by the class counselors. The entire survey took about 15 min. After eliminating 45 invalid questionnaires, because the answering time was too short (i.e., completed in < 120 s), finally 555 valid questionnaires were collected. The present study was carried out in accordance with the Declaration of Helsinki (Goodyear et al., 2007), and all participants voluntarily filled out questionnaires and signed informed consent.

### Measures

There were 44 items in the final questionnaire, consisting of three constructs, i.e., EOI, PC, and EI, and three demographic factors of the respondents, i.e., gender, grade, and major. The items of EOI, PC, and EI were all measured on a 5-point Likert-type scale, ranging from 1 (strongly disagree) to 5 (strongly agree).

### Entrepreneurial intention

Entrepreneurial Intention was assessed using a measurement developed by Iakovleva et al. (2011) in this study. This scale consists of 10 items containing two dimensions: three items for entrepreneurial goal intention and seven items for goal implementation intention, the higher scores suggesting higher levels of EI. The sample item is “My professional goal is to become an entrepreneur.”

### Psychological capital

To measure PC, a short version of the scale developed by Luthans et al. (2007a) was used for this study. This short version scale comprised 16 items and four dimensions: self-efficacy, optimism, hope, and resilience. The higher the score, the higher the level of PC. The sample item is “When things are uncertain for me, I usually expect the best.”

### Entrepreneurial opportunity identification

To measure EOI, a creativity-based theoretical scale proposed by Hansen et al. (2011) was used. This scale consists of 15 items and five dimensions: preparation, incubation, insight, evaluation, and elaboration. Each dimension of this scale has three items, the higher the score indicates the higher the level of EOI. The sample item is “The most important thing is to believe in the idea.”

### Model-data fit of the measurement model

Confirmatory factor analysis (CFA) was performed with AMOS software to ensure that the measurement models have good structural validity. Since an enormous sample size may cause increased chi-square values (the value is greater than the recommended value, i.e., < 5) (Doll et al., 1994), the following indices are considered to test the fit indices of measurement models: RMR(< 0.05), RMSEA (< 0.08), CFI (>0.90), GFI (>0.90), TLI (>0.90), NFI (>0.90), IFI (>0.90), and SRMR(< 0.05) (Hu and Bentler, 1999; McDonald and Ho, 2002; Wolf et al., 2013; Hayes, 2015; Pavlov et al., 2021). CFA results are shown in Table 1. As a whole, the results indicated a reasonable model-data fit of the measurement models.

**Table 1:** Measurement model validity.

	**X^2^/df**	**RMR**	**RMSEA**	**CFI**	**GFI**	**TLI**	**NFI**	**IFI**	**SRMR**
EOI	3.087	0.024	0.061	0.940	0.944	0.921	0.914	0.940	0.044
PC	3.407	0.033	0.066	0.927	0.927	0.911	0.901	0.928	0.052
EI	6.120	0.034	0.096	0.925	0.928	0.900	0.912	0.925	0.052

EOI, entrepreneurial opportunity identification; PC, psychological capital; EI, entrepreneurial intention; CFI, comparative fit index; GFI, goodness-of-fit index; TLI, Tucker–Lewis index; NFI, normed fit index; IFI, incremental fit index; SRMR, standardized root mean residual.

For the confirmation of the reliability of the constructs, the average variance extracted (AVE), construct reliability (CR) of each dimension of the scales, and Cronbach's alpha (α) were computed. As shown in Table 2, where the minimum of AVE is above 0.36, Cronbach's α is more than 0.7, CR is more than the standard of 0.6, and the average factor loading of all items is basically larger than 0.6 and overpass 0.5, suggesting that all dimensions of the constructs have a good acceptable convergent validity (Fornell and Larcker, 1981).

**Table 2:** Reliability of constructs.

**Variable name**	**No. of items**	**Avg CFA loading**	**CR**	**AVE**	**Alpha**
EOI-preparation	3	0.630	0.666	0.402	0.878
EOI-incubation	3	0.741	0.786	0.550	
EOI-insight	3	0.661	0.701	0.442	
EOI-evaluation	3	0.596	0.624	0.360	
EOI-elaboration	3	0.723	0.768	0.525	
PC-self-efficacy	4	0.733	0.823	0.539	0.892
PC-optimism	4	0.612	0.709	0.386	
PC-hope	4	0.634	0.733	0.414	
PC-resilience	4	0.684	0.779	0.469	
EI-entrepreneurial goal intention	3	0.718	0.773	0.545	0.874
EI-goal implementation intention	7	0.655	0.844	0.444	

EOI, entrepreneurial opportunity identification; PC, psychological capital; EI, entrepreneurial intention.

As to the divergent validity, the method of the squared root of AVE recommended by Fornell and Larcker (1981) was used. As displayed in Table 3, the number of squared roots of AVE of every dimension is found larger than the coefficient correlation with every construct, thus meeting the criteria for evaluation divergent validity.

**Table 3:** Discriminant validity.

	**M**	**SD**	**PC-1**	**PC-2**	**PC-3**	**PC-4**	**EOI-1**	**EOI-2**	**EOI-3**	**EOI-4**	**EOI-5**	**EI-1**	**EI-2**
PC-1	3.140	0.621	**0.734**										
PC-2	3.128	0.620	0.583^***^	**0.621**									
PC-3	3.334	0.589	0.586^***^	0.535^***^	**0.643**								
PC-4	3.299	0.591	0.462^***^	0.482^***^	0.627^***^	**0.685**							
EOI-1	3.585	0.535	0.421^***^	0.360^***^	0.465^***^	0.442^***^	**0.634**						
EOI-2	3.505	0.594	0.499^***^	0.400^***^	0.517^***^	0.479^***^	0.635^***^	**0.742**					
EOI-3	3.348	0.624	0.337^***^	0.374^***^	0.373^***^	0.324^***^	0.426^***^	0.546^***^	**0.665**				
EOI-4	3.476	0.592	0.363^***^	0.353^***^	0.289^***^	0.301^***^	0.462^***^	0.442^***^	0.389^***^	**0.600**			
EOI-5	3.590	0.558	0.401^***^	0.363^***^	0.397^***^	0.400^***^	0.525^***^	0.494^***^	0.450^***^	0.545^***^	**0.725**		
EI-1	3.136	0.640	0.539^***^	0.402^***^	0.349^***^	0.345^***^	0.267^***^	0.354^***^	0.235^***^	0.234^***^	0.234^***^	**0.738**	
EI-2	3.045	0.582	0.643^***^	0.448^***^	0.405^***^	0.349^***^	0.353^***^	0.459^***^	0.368^***^	0.311^***^	0.368^***^	0.690^***^	**0.666**

EOI-1, preparation; EOI-2, incubation; EOI-3, insight; EOI-4, evaluation; EOI-5, elaboration; PC-1, self-efficacy; PC-2, optimism; PC-3, hope; PC-4, resilience; EI-1, entrepreneurial goal intention; EI-2, goal implementation intention. The bold is the squared root of AVE. ^***^p < 0.001.

### Data analysis

Before statistical analysis, Harman's single factor was used to assess the common method variance. Second, respondents' demographic profiles, descriptive statistics, and correlation analysis were performed using SPSS 21.0 software. Third, to assess the hypothesis, the covariance base structural equation modeling (CB-SEM) in AMOS 21.0 software was used, not least because it could test relationships between many factors simultaneously (Kline, 1988). Finally, to test the vigor of the mediating effect, the method of bootstrapping with 5,000 times resampling was further conducted. According to Preacher and Hayes (2008), compared with the traditional causal steps, the method of bootstrapping has shown greater statistical power.

## Results

### Common method variance test

In self-report surveys, there may be common method variance with the potential to postulate relations in a model. In the present study, Harman's single factor test was performed to check for common method variance (Podsakoff and Organ, 1986). A total of seven factors with an eigenvalue over one were obtained by unrotated principal component factor analysis for all variables. The first emerging unrotated factor accounted for 29.898% of the overall, lower than the standard value of 50% (Podsakoff et al., 2003). The obtained data could be further analyzed.

### Respondents' profile

Table 4 shows the respondents' profiles. For gender, a majority of respondents were women (445, 80.2%), whereas 110 (19.8%) were men. For the grade, 100 respondents (18%) were freshmen, 102 respondents (18.4%) were sophomores, 294 respondents (53%) were juniors, and 59 respondents (10.6%) were seniors. For respondents' majors, 456 respondents (82.2%) were humanities, 73 respondents (13.2%) were science, and 26 respondents (4.7%) were engineering.

**Table 4:** Profile of participants.

**Item**	**Category**	**Frequency**	**Percent**
Gender	Male	110	19.8%
	Female	445	80.2%
Grade	Freshman	100	18%
	Sophomore	102	18.4%
	Junior	294	53%
	Senior	59	10.6%
Major	Humanities	456	82.2%
	Science	73	13.2%
	Engineering	26	4.7%

### Descriptive statistics and correlation analysis

Descriptive statistics and correlations of the main variables are displayed in Table 5. The results show the EOI (M = 3.501), EI (M = 3.072), and PC (M = 3.225) of university students. It can be seen that university students' EOI, EI, and PC were at an above average level. University students' EOI was significantly correlated with PC (*r* = 0.627, *p* < 0.001), and EI was significantly correlated with PC (*r* = 0.591, *p* < 0.001); in addition, EOI was significantly correlated with EI (*r* = 0.472, *p* < 0.001). Moreover, the correlation coefficients between the variables ranged from 0.472 to 0.627, all of which were significant but not more than 0.8, showing that there were no high correlations and no serious collinearity problems.

**Table 5:** Descriptive statistics and correlations among variables.

**Variables**	**M**	**SD**	**EOI**	**PC**	**EI**
EOI	3.501	0.447	1		
PC	3.225	0.491	0.627^***^	1	
EI	3.072	0.558	0.472^***^	0.591^***^	1

^***^p < 0.001; M, mean; SD, standard deviation; EOI, entrepreneurial opportunity identification; PC, psychological capital; EI, entrepreneurial intention.

### Test of hypothesis

In the present study, ANOVA analysis and *t*-test results indicate that gender (*t* = 0.482, *p* > 0.050), grade (*F* = 2.446, *p* > 0.050), and major (*F* = 1.598, *p* > 0.050) have no significant difference in university students' EI. Therefore, this research does not control the impact on EI of gender, grade, and major. This study hypothesized EOI as the predictive variable, EI as the dependent variable, and PC as the mediating variable of the relation between EOI and EI. Two models of SEM in AMOS 21.0 were constructed as follows.

First, this research adopted model 1 to construct the main effect model of the impact of EOI on EI. The model fitness index is shown in Table 6, which means that this structural model has a reasonable model-data fit (Hu and Bentler, 1999; Wolf et al., 2013; Hayes, 2015; Pavlov et al., 2021).

**Table 6:** Structural model validity.

**Models**	**X^2^/df**	**RMR**	**RMSEA**	**CFI**	**GFI**	**TLI**	**NFI**	**IFI**	**SRMR**
Model 1: EOI → EI	5.175	0.012	0.087	0.963	0.966	0.941	0.955	0.964	0.035
Model 2: EOI → PC → EI	5.864	0.016	0.094	0.929	0.924	0.905	0.916	0.929	0.045

EOI, entrepreneurial opportunity identification; EI, entrepreneurial intention; PC, psychological capital; CFI, comparative fit index; GFI, goodness-of-fit index; TLI, Tucker–Lewis index; NFI, normed fit index; IFI, incremental fit index; SRMR, standardized root mean residual.

**Figure 2:**
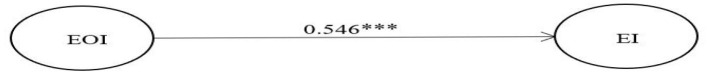
The effects of EOI on EI. ***p < 0.001; EOI, entrepreneurial opportunity identification; EI, entrepreneurial intention.

According to the research result (as in Figure 2), EOI can significantly predict university students' EI (β = 0.546, *p* < 0.001), so H1 is supported.

**Figure 3:**
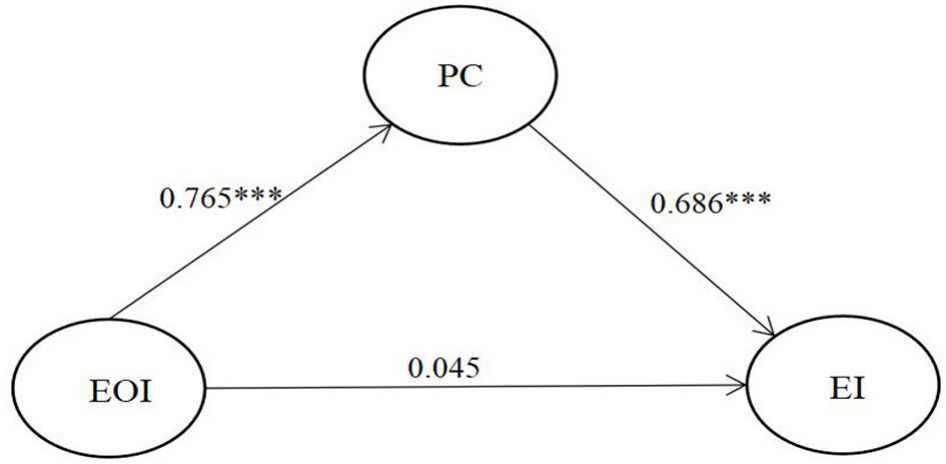
Testing the mediation model. ***p < 0.001; EOI, entrepreneurial opportunity identification; PC, psychological capital; EI, entrepreneurial intention.

Second, the present study adopted model 2 to examine the mediating effect of PC on EOI and EI. As per the test procedures for mediating effect (Nevitt and Hancock, 2001; Preacher and Hayes, 2008; Hayes, 2018), PC was added as the mediating variable between EOI and EI. The model analysis results are shown in Table 6, indicating that the mediating effect had a reasonable model-data fit (Hu and Bentler, 1999; McDonald and Ho, 2002; Wolf et al., 2013; Hayes, 2015; Pavlov et al., 2021). According to the research results (as in Figure 3), in the path of EOI → PC → EI, EOI has a significant and positive impact on PC (β = 0.765, *p* < 0.001); PC has a significant and positive impact on EI (β = 0.686, *p* < 0.001); EOI does not have a significant impact on EI (β = 0.045, *p* > 0.05). It can be seen that PC can significantly predict college students' EIs, so H2 is supported. However, after adding PC as the mediating variable between EOI and EI, the path coefficient of the impact of EOI on EI is not significant anymore. This suggests that PC capital plays a fully mediating role between EOI and EI, supporting H3.

The method of bootstrapping that randomly repeated sampling 5,000 times with AMOS 21.0 was further used to test the stability of the mediating model. The results are shown in Table 7.

**Table 7:** Bootstrap analyses of a hypothesis mediation model.

**Effects**	**β**	***p***	**95% confidence interval**	
			**Lower limit**	**Upper limit**
**Direct effect**				
EOI → PC	0.765^***^	< 0.001	0.695	0.826
PC → EI	0.686^***^	< 0.001	0.501	0.851
EOI → EI	0.045	=0.559	−0.120	0.213
**Indirect effect**				
EOI → CP → EI	0.525^***^	< 0.001	0.383	0.673
Total effect	0.570***	< 0.001	0.493	0.645

^***^p < 0.001; EOI, entrepreneurial opportunity identification; PC, psychological capital; EI, entrepreneurial intention.

As shown in Table 7, the direct effect of EOI on PC on EI was 0.765, and the 95% CI (0.695–0.826) excluded 0; the direct effect of EOI and PC was 0.686, the 95% CI (0.501–0.851) excluded 0; however, the direct effect of EOI on EI was 0.045, and the 95% confidence interval (−0.120 to 0.213) included 0. This implied that in the mediating model, the effect of EOI on EI was not significant, PC played a full mediating role between EOI on entrepreneurial, the effect of mediation (0.765 ^*^ 0.686) was 0.525, and the 95% confidence (0.383–0.673) interval excluded 0. Moreover, the total effect was 0.570. These findings show that the mediation effect of PC works well in our proposed model. Without PC, university student's sense and ability of EOI cannot exert a significant effect on their EI.

## Discussion

### Main findings

Drawing upon the EEM and previous studies on EI, in a sample of 555 university students in China, the present study aimed to examine the role of EOI and PC in developing EI among university students.

First, the present study shows that university students' EOI significantly and positively affected their EI, supporting H1. This result is similar to the previous results conducted in the field of business (e.g., Zahra et al., 2009; Hills et al., 2011; Mahmood et al., 2019), indicating that the higher the level of university students' EOI is, the higher their EI will be. That is, university students' sense and ability of EOI is an important predictor of EI. Having a favorable attitude toward entrepreneurship does not necessarily form EI. Only when identifying a potential business opportunity, university students could show up a higher level of their EI thus engaging in starting an entrepreneurial journey.

As far as the nature of the entrepreneurial opportunity, there are two competing views, i.e., the discovery and the creation point of view (Edelman and Yli-Renko, 2010; Kuckertz et al., 2017; Niu et al., 2022). Moreover, yet for university students, the creation point of view is more appropriate than the discovery point of view for examining university students' ability of EOI (Munoz et al., 2011; Chen and Tao, 2021). Since they have no direct business experiences, no exposure to the changing market and the multiply information, so entrepreneurial opportunity is not out there, merely wait to be discovered by university students (Hansen et al., 2011). One of the main effects resulting from entrepreneurial education, however, is to enhance the creativity of university students. Scholars have noticed that entrepreneurial education could positively and significantly affect creativity (Hu et al., 2018; Shi et al., 2020). According to Alshebami et al. (2022), for instance, lecturers' creativity could significantly influence students' entrepreneurial intentions. In this sense, considering the creativity-related nature of the entrepreneurial opportunity, developing the identification of opportunities is essential for entrepreneurial education programs.

Second, according to the H2, PC has a positive prediction of university students' EI, that is, enhancing university students' PC is key to improving their lower EI. This result is in line with previous research that the psychological resources appropriate for PC are linked to entrepreneurship (Baron et al., 2016; Nolzen, 2018; Wang et al., 2022). This study, with inspiration from Ephrem et al. (2019) and Mahfud et al. (2020), emphasizes the importance of PC in explaining why some individuals are more willing toward entrepreneurship than others. Compared with university students' EOI, the present study found that university students' PC had a greater effect on their EI, and deeply understanding the psychological factors of university students is especially important when university students have perceived the feasibility of a new business.

In the present study, PC was regarded as a conceptual superstructure, separately from each component (Luthans et al., 2007a). Increasing the PC of university students will help them define their personal career goals, develop their self-belief to achieve those goals, and overcome the fear of entrepreneurship (Baluku et al., 2016). Those students with higher PC, i.e., have the skills to have faith in their skills and insights, to identify business opportunities where others see disorder, to focus on different ways to achieve their goals, and to get back up from a fall and adversity, are more engaged in courageous behaviors than students who do not.

Third, according to the H3, this research further discovered that PC positively mediates the relationship between EOI and EI. The finding is in line with previous studies on the TPB (Karimi and Makreet, 2020), which supported the mediating role of psychological factors in the relationship between EOI and EI. Consistent with those studies, this study further reinforces that EI requires not only strong desirability and personal feasibility, shown in a sense and ability toward entrepreneurial opportunity, but also needs to strengthen psychological resources. Moreover, our study shows that PC mediates the full effect of EOI on EI. University students' lower sense and ability of EOI need to be counterbalanced with higher levels of psychological investment, that is, EOI alone cannot influence the intention of university students' EI.

The full mediating effect of PC on EI was also reported by some recent studies. For example, recently, in a sample of 215 polytechnic students in Indonesia, Mahfud et al. (2020) found that entrepreneurial attitude orientation, social capital, and PC could collaboratively influence the polytechnic students' EI, and PC was found to fully mediate the impact of social capital on EI. Similarly, a study targeted 752 female students, and Chang et al. (2022) indicated that an entrepreneurial mindset had a mediation effect between entrepreneurial competency and EI; in particular, without an entrepreneurial mindset, entrepreneurial competency alone cannot significantly exert effects on EI. As with other research, this finding from the present study was not conclusive; to understand this issue, more research should be added up.

### Theoretical contributions and practical implications

The present result may provide some theoretical contributions to the literature. First, although previous research in the field of business has confirmed that EOI has a significant and positive impact on entrepreneurship intention, the influence of EOI on entrepreneurship intention and its underlying mechanism remains unclear. The present study explored the mediating mechanism of EOI on EI in the field of education, indicating that PC plays an important mediating role in university students' entrepreneurial intent. The result could enrich previous understanding about the relationship between EOI and EI in the field of business, adding further empirical evidence to entrepreneurship literature. Second, the present study followed Liñán and Fayolle (2015) previous suggestions, according to them, researching EI should transfer from traditional models, which mainly focus on the independent and direct explanatory variables, to an approach of indirect factors. In this vein, the present study highlighted the key indirect role of PC. Especially, PC has a full mediating role in the relationship between EOI and EI. This study result could add further empirical evidence to the “core structure” of PC in the field of education, i.e., collective PC has a stronger effect than that of each of its components (Luthans et al., 2007a). Compared with university students' sense and ability of EOI, the development of university students' PC is more important to cultivate their EI.

Some practical suggestions could also be inferred from the present study. It is recommended that entrepreneurial courses and training programs carried out by educators should concentrate on cultivating cognitive factors; among which, educators and practitioners should pay regard to the role of EOI and PC collaboratively. On the one hand, educators should design strategies to increase university students' sense and ability of EOI, which can be addressed by experience-based learning processes, such as hands-on activities, simulation case studies, and business competition (Wang and Ortiz, 2022). On the other hand, having the necessary skills of EOI to initiate entrepreneurial activities alone is not necessarily leading to university students' entrepreneurial behaviors; educators need to equip students with positive psychological resources, such as PC, which can be developed and taught. Various measures could be adopted by educators to improve students' levels of PC, such as local case studies and face-to-face discussions with successful entrepreneurs (Su et al., 2020; Xie et al., 2022). The training and learning process of PC could positively affect students' EI by increasing their level of creativity, information processing ability, and intellectual fluency, which, in turn, enhances the chances to act upon identified business opportunities (Ephrem et al., 2019). PC can be developed for university students, the most potential group of entrepreneurs.

### Limitations and future research

Some limitations must be noted in the present study, which could be carried out in future studies. First, following previous studies, data used in this study were collected only from one university in China with a cross-sectional design; thus, a general conclusion cannot be made. Especially, although EI may be the predictor of entrepreneurship, it is not the behavior itself (Neneh, 2019), and the level of EI may change over time. To generalize the conclusion, future studies would use longitudinal data to track the intention-behavior process. Such studies could look closer at which factors impact the transformation of intention into actions (Li et al., 2020).

Another limitation is that PC was collected among university students, and the measure of which was used by a general version of PC, not entrepreneurial PC *per se*. University students might have different PC compared with other groups, such as nascent entrepreneurs (Bockorny and Youssef-Morgan, 2019; Chen and Tao, 2021). In this vein, future studies should collect data from young entrepreneurs with the entrepreneurial PC scale, to compare the PC of entrepreneurs to that of would-be entrepreneurs.

Finally, in this study, only PC was used as the mediator of EOI to draw inferences on EI, there are other cognitive variables that could act as a mediator. Thus, more cognitive factors (e.g., locus of control, behavioral attitude, and subjective social norms) and psychological resources (e.g., autonomy, entrepreneurial competence, and positive emotions) can be used in future studies (Baluku et al., 2018; Chen et al., 2021; Lv et al., 2021).

## Conclusion

Based on the entrepreneurial event model (EEM), the present study tried to propose a mediating model for understanding the role of PC in the relationship between EOI and EI among university students from the positive psychology perspective. The results concluded that university students' sense and ability of EOI had a positive and significant effect on their EI. In particular, the mediating role of PC between university students' EOI and their entrepreneurship intentions was found. Enhancing the PC of university students could increase their capacity to use entrepreneurship opportunities and develop higher intentions for entrepreneurial initiatives. The present study could shed light on new instructions to examine the interaction between the cognitive and psychological components of entrepreneurship. Considering the importance of the entrepreneurial opportunity to sustainable entrepreneurship, more mechanisms between EOI and EI should be carried out in future studies.

## Data availability statement

The original contributions presented in the study are included in the article/supplementary material, further inquiries can be directed to the corresponding authors.

## Ethics statement

Written informed consent was obtained from the individual(s), and minor(s)' legal guardian/next of kin, for the publication of any potentially identifiable images or data included in this article.

## Author contributions

HZ and HC designed the study, revised the manuscript, collected, and analyzed the data. HZ provided the original manuscript. Both authors contributed to the article and approved the submitted version.

## References

[B1] Abdelwahed N. A. A. (2022). Developing entrepreneurial sustainability among saudi arabia's university students. Sustainability 14, 11890. 10.3390/su14191189035465476

[B2] Ajzen I. (1991). The theory of planned behavior. Organ. Behav. Hum. Decis. Process. 50, 179–211. 10.1016/0749-5978(91)90020-T

[B3] Al-Harasi A. H. Surin E. F. Rahim H. L. Al-Shammari S. A. Abdulrab M. Al-Mamary Y. H.. (2021). The impact of social entrepreneurial personality on social entrepreneurial intention among university graduates in Yemen: a conceptual framework. Holos 37, 1–17. 10.15628/holos.2021.11420

[B4] Alshebami A. S. (2021). The influence of psychological capital on employees' innovative behavior: mediating role of employees' innovative intention and employees' job satisfaction. SAGE Open 11. 10.1177/2158244021104080935661464

[B5] Alshebami A. S. Seraj A. H. A. Alzain E. (2022). Lecturers' creativity and students' entrepreneurial intention in Saudi Arabia. Vision. 10.1177/09722629221099596

[B6] Antončič B. Auer Antončič J. (2023). Psychological and sociological determinants of entrepreneurial intentions and behaviors. Front. Psychol. 14, 1076768. 10.3389/fpsyg.2023.1076768 36818098 PMC9932979

[B7] Bae T. J. Qian S. Miao C. Fiet J. O. (2014). The relationship between entrepreneurship education and entrepreneurial intentions: a meta-analytic review. Entrep. Theory Pract. 38, 217–254. 10.1111/etap.12095

[B8] Baluku M. Kikooma J. Kibanja G. (2016). Psychological capital and the startup capital-entrepreneurial success relationship. J. Small Bus. Entrep. 28, 27–54. 10.1080/08276331.2015.1132512

[B9] Baluku M. M. Kikooma J. F. Otto K. (2018). Positive mindset and entrepreneurial outcomes: the magical contributions of psychological resources and autonomy. J. Small Bus. Entrep. 30, 473–498. 10.1080/08276331.2018.1459017

[B10] Bao J. Zhou X. Chen Y. (2017). Entrepreneurial passion and behaviors: opportunity recognition as a mediator. Soc. Behav. Pers. Int. J. 45, 1211–1220. 10.2224/sbp.6492

[B11] Baron R. A. Franklin R. J. Hmieleski K. M. (2016). Why entrepreneurs often experience low, not high levels of stress: the joint effects of selection and psychological capital. J. Manag. 42, 742–768. 10.1177/014920631349541135220971

[B12] Bazkiaei H. A. Heng L. H. Khan N. U. Saufi R. B. A. Kasim R. S. R. (2020). Do entrepreneurial education and big-five personality traits predict entrepreneurial intention among universities students? Cogent Bus. Manage. 7, 1801217. 10.1080/23311975.2020.1801217

[B13] Bockorny K. Youssef-Morgan C. M. (2019). Entrepreneurs' courage, psychological capital, and life satisfaction. Front. Psychol. 10, 789. 10.3389/fpsyg.2019.00789 31024410 PMC6461011

[B14] Chang A. Chang D.-F. Chen T.-L. (2022). Detecting female students transforming entrepreneurial competency, mindset, and intention into sustainable entrepreneurship. Sustainability 14, 12970. 10.3390/su142012970

[B15] Chen B.-S. Yuan C.-H. Yin B. Wu X.-Z. (2021). Positive emotions and entrepreneurial intention: the mediating role of entrepreneurial cognition. Front. Psychol. 12, 760328. 10.3389/fpsyg.2021.760328 34867660 PMC8632803

[B16] Chen H. Tao Y. (2021). Efficacy of entrepreneurs' psychological capital on the performance of new ventures in the development of regional economy in the greater bay area. Front. Psychol. 12, 705095. 10.3389/fpsyg.2021.705095 34650473 PMC8510642

[B17] Choi J. Hwang K. (2020). The effects of nascent Entrepreneurs' positive psychological capital and public self-consciousness on entrepreneurial intentions. J. Inform. Technol. Appl. Manag. 27, 15–47. 10.21219/jitam.2020.27.1.015

[B18] Contreras F. Dreu I. Espinosa J. C. (2017). Examining the relationship between psychological capital and entrepreneurial intention: an exploratory study. Asian Soc. Sci. 13, 80–88. 10.5539/ass.v13n3p80

[B19] Corner P. D. Ho M. (2010). How opportunities develop in social entrepreneurship. Entrep. Theory Pract. 34, 635–659. 10.1111/j.1540-6520.2010.00382.x

[B20] Dheer R. J. Lenartowicz T. (2018). Multiculturalism and entrepreneurial intentions: understanding the mediating role of cognitions. Entrep. Theory Pract. 42, 426–466. 10.1111/etap.12260

[B21] Digan S. P. Sahi G. K. Mantok S. Patel P. C. (2019). Women's perceived empowerment in entrepreneurial efforts: the role of bricolage and psychological capital. J. Small Bus. Manag. 57, 206–229. 10.1111/jsbm.12402

[B22] Doll W. J. Xia W. Torkzadeh G. (1994). A confirmatory factor analysis of the end-user computing satisfaction instrument. MIS Q. 18, 453–461. 10.2307/249524

[B23] Donaldson C. (2019). Intentions resurrected: a systematic review of entrepreneurial intention research from 2014 to 2018 and future research agenda. Int. Entrep. Manag. J. 15, 953–975. 10.1007/s11365-019-00578-5

[B24] Edelman L. Yli-Renko H. (2010). The impact of environment and entrepreneurial perceptions on venture-creation efforts: bridging the discovery and creation views of entrepreneurship. Entrep. Theory Pract. 34, 833–856. 10.1111/j.1540-6520.2010.00395.x

[B25] Ephrem A. N. Namatovu R. Basalirwa E. M. (2019). Perceived social norms, psychological capital and entrepreneurial intention among undergraduate students in Bukavu. Educ. Train. 61, 963–983. 10.1108/ET-10-2018-0212

[B26] Fishbein M. Ajzen I. (2011). Predicting and Changing Behavior: The Reasoned Action Approach. Hove: Psychology Press.

[B27] Fornell C. Larcker D. F. (1981). Evaluating structural equation models with unobservable variables and measurement error. J. Mark. Res. 18, 39–50. 10.1177/002224378101800104

[B28] Galvao A. Marques C. S. Marques C. P. (2018). Antecedents of entrepreneurial intentions among students in vocational training programmes. Educ. Train. 60, 719–734. 10.1108/ET-03-2017-0034

[B29] Gao Y. Qin X. (2022). Entrepreneurship education and entrepreneurial intention of Chinese college students: evidence from a moderated multi-mediation model. Front. Psychol. 13, 1049232. 10.3389/fpsyg.2022.1049232 36524159 PMC9745182

[B30] Goodyear M. D. Krleza-Jeric K. Lemmens T. (2007). The declaration of Helsinki. BMJ 335, 624–625. 10.1136/bmj.39339.610000.BE 17901471 PMC1995496

[B31] Grégoire D. A. Shepherd D. A. Schurer Lambert L. (2010). Measuring opportunity-recognition beliefs: illustrating and validating an experimental approach. Organ. Res. Methods 13, 114–145. 10.1177/1094428109334369

[B32] Guo R. Yin H. Lv X. (2022). Improvisation and university students' entrepreneurial intention in China: the roles of entrepreneurial self-efficacy and entrepreneurial policy support. Front. Psychol. 13, 930682. 10.3389/fpsyg.2022.930682 36072027 PMC9441934

[B33] Hanohov R. Baldacchino L. (2017). Opportunity recognition in sustainable entrepreneurship: an exploratory study. Int. J. Entrep. Behav. Res. 24, 333–358. 10.1108/IJEBR-12-2015-0275

[B34] Hansen D. J. Lumpkin G. T. Hills G. E. (2011). A multidimensional examination of a creativity-based opportunity recognition model. Int. J. Entrep. Behav. Res. 17, 515–533. 10.1108/13552551111158835

[B35] Hassan A. Saleem I. Anwar I. Hussain S. A. (2020). Entrepreneurial intention of Indian university students: the role of opportunity recognition and entrepreneurship education. Educ. Train. 62, 843–861. 10.1108/ET-02-2020-0033

[B36] Hayes A. F. (2015). An index and test of linear moderated mediation. Multivariate Behav. Res. 50, 1–22. 10.1080/00273171.2014.962683 26609740

[B37] Hayes A. F. (2018). Introduction to Mediation, Moderation, and Conditional Process Analysis: A Regession Based-Approach. 2nd Edn. New York, NY: Guilford Press.

[B38] Hills G. E. Singh R. P. Lumpkin G. T. Baltrusaityte-Axelson J. (2011). Opportunity Recognition: Examining How Search Formality and Search Processes Relate to the Reasons for Pursuing Entrepreneurship. Rochester, NY: Social Science Electronic Publishing.

[B39] Hoang G. Luu T. T. Le T. T. T. Tran A. K. T. (2022). Dark Triad traits affecting entrepreneurial intentions: the roles of opportunity recognition and locus of control. J. Bus. Vent. Insights 1, e00310. 10.1016/j.jbvi.2022.e00310

[B40] Hou F. Su Y. Qi M. Chen J. Tang J. (2022). A multilevel model of entrepreneurship education and entrepreneurial intention: opportunity recognition as a mediator and entrepreneurial learning as a moderator. Front. Psychol. 13, 837388. 10.3389/fpsyg.2022.837388 35222214 PMC8873978

[B41] Hu L. T. Bentler P. M. (1999). Cutoff criteria for fit indexes in covariance structure analysis: conventional criteria versus new alternatives. Struct. Equ. Model. Multidiscip. J. 6, 1–55. 10.1080/1070551990954011836787513

[B42] Hu R. Wang L. Zhang W. Bin P. (2018). Creativity, proactive personality, and entrepreneurial intention: the role of entrepreneurial alertness. Front. Psychol. 9:951. 10.3389/fpsyg.2018.00951 29962985 PMC6011088

[B43] Iakovleva T. Kolvereid L. Stephan U. (2011). Entrepreneurial intentions in developing and developed countries. Educ. Train. 53, 353–370. 10.1108/00400911111147686

[B44] Karimi S. Biemans H. J. NaderiMahdei K. Lans T. Chizari M. Mulder M. (2017). Testing the relationship between personality characteristics, contextual factors and entrepreneurial intentions in a developing country. Int. J. Psychol. 52, 227–240. 10.1002/ijop.12209 26334129

[B45] Karimi S. Makreet A. S. (2020). The role of personal values in forming students' entrepreneurial intentions in developing countries. Front. Psychol. 11, 525844. 10.3389/fpsyg.2020.525844 33329168 PMC7710526

[B46] Kirzner I. M. (1979). Perception, Opportunity, and Profit: Studies in the Theory of Entrepreneurship. Chicago, IL: University of Chicago Press.

[B47] Kline R. B. (1988). Principles and Practice of Structural Equation Modeling. New York, NY: Guilford.

[B48] Krueger N. F. (2007). The Cognitive Infrastructure of Opportunity Emergence in Entrepreneurship. Heidelberg: Springer.

[B49] Kuckertz A. Kollmann T. Krell P. Stöckmann C. (2017). Understanding, differentiating, and measuring opportunity recognition and opportunity exploitation. Int. J. Entrep. Behav. Res. 23, 78–97. 10.1108/IJEBR-12-2015-029025467446

[B50] Lent R. W. Lopez F. G. Bieschke K. J. (1993). Predicting mathematics-related choice and success behaviors: test of an expanded social cognitive model. J. Vocat. Behav. 42, 223–236. 10.1006/jvbe.1993.1016

[B51] Li C. Murad M. Shahzad F. Khan M. A. S. Ashraf S. F. Dogbe C. S. K. (2020). Entrepreneurial passion to entrepreneurial behavior: role of entrepreneurial alertness, entrepreneurial self-efficacy and proactive personality. Front. Psychol. 11, 1611. 10.3389/fpsyg.2020.01611 32973593 PMC7468520

[B52] Lin L. Lin Y. Lin S. (2021). The journey of business opportunity evaluation: when and why does opportunity novelty promote vs. inhibit opportunity adoption? Front. Psychol. 12, 732565. 10.3389/fpsyg.2021.732565 34712179 PMC8545874

[B53] Liñán F. Fayolle A. (2015). A systematic literature review on entrepreneurial intentions: citation, thematic analyses, and research agenda. Int. Entrep. Manag. J. 11, 907–933. 10.1007/s11365-015-0356-5

[B54] Liñán F. Rodríguez-Cohard J. C. Rueda-Cantuche J. M. (2011). Factors affecting entrepreneurial intention levels: a role for education. Int. Entrep. Manag. J. 7, 195–218. 10.1007/s11365-010-0154-z

[B55] Luo Y.-F. Huang J. Gao S. (2022). Relationship between proactive personality and entrepreneurial intentions in college students: mediation effects of social capital and human capital. Front. Psychol. 13, 861447. 10.3389/fpsyg.2022.861447 35783804 PMC9243360

[B56] Luthans F. Avolio B. J. Avey J. B. (2007a). Psychological capital (PsyCap) questionnaire (PCQ). Menlo Park, CA: Mind Garden, Inc.

[B57] Luthans F. Avolio B. J. Avey J. B. Norman S. M. (2007b). Positive psychological capital: measurement and relationship with performance and satisfaction. Pers. Psychol. 60, 541–572. 10.1111/j.1744-6570.2007.00083.x

[B58] Lv Y. Chen Y. Sha Y. Wang J. An L. Chen T.. (2021). How entrepreneurship education at universities influences entrepreneurial intention: mediating effect based on entrepreneurial competence. Front. Psychol. 12, 655868. 10.3389/fpsyg.2021.655868 34295281 PMC8289882

[B59] Mahfud T. Triyono M. B. Sudira P. Mulyani Y. (2020). The influence of social capital and entrepreneurial attitude orientation on entrepreneurial intentions: the mediating role of psychological capital. Eur. Res. Manag. Bus. Econ. 26, 33–39. 10.1016/j.iedeen.2019.12.005

[B60] Mahmood T. M. A. T. Al Mamun A. Ahmad G. B. Ibrahim M. D. (2019). Predicting entrepreneurial intentions and pre-start-up behavior among *Asnaf millennials*. Sustainability 11, 4939. 10.3390/su11184939

[B61] Maslakci A. Sesen H. Sürücü L. (2021). Multiculturalism, positive psychological capital and students' entrepreneurial intentions. Educ. Train. 63, 597–612. 10.1108/ET-04-2020-0073

[B62] McDonald R. P. Ho M. H. R. (2002). Principles and practice in reporting structural equation analyses. Psychol. Methods 7, 64–82. 10.1037/1082-989X.7.1.64 11928891

[B63] Munoz A. Mosey S. Binks M. (2011). Developing opportunity identification capabilities in the classroom: visual evidence for changing mental frames. Acad. Manag. Learn. Educ. 10, 277–295. 10.5465/amle.10.2.zqr277

[B64] Na B. Ahmad N. H. Zhang C. Han Y. (2022). Entrepreneurial intention and delayed job satisfaction from the perspective of emotional interaction: the mediating of psychological capital. Front. Psychol. 13, 925460. 10.3389/fpsyg.2022.925460 35774949 PMC9239561

[B65] Ndofirepi T. M. (2020). Relationship between entrepreneurship education and entrepreneurial goal intentions: psychological traits as mediators. J. Innov. Entrepr. 9, 1–20. 10.1186/s13731-020-0115-x

[B66] Neneh B. N. (2019). From entrepreneurial intentions to behavior: the role of anticipated regret and proactive personality. J. Vocat. Behav. 112, 311–324. 10.1016/j.jvb.2019.04.00535734452

[B67] Nevitt J. Hancock G. R. (2001). Performance of bootstrapping approaches to model test statistics and parameter standard error estimation in structural equation modeling. Struct. Equ. Model. 8, 353–377. 10.1207/S15328007SEM0803_2

[B68] Niu X. Niu Z. Wang M. Wu X. (2022). What are the key drivers to promote entrepreneurial intention of vocational college students? An empirical study based on structural equation modeling. Front. Psychol. 13, 1021969. 10.3389/fpsyg.2022.1021969 36389516 PMC9650398

[B69] Nolzen N. (2018). The concept of psychological capital: a comprehensive review. Manag. Rev. Q. 68, 237–277. 10.1007/s11301-018-0138-6

[B70] Ozgen E. Baron R. A. (2007). Social sources of information in opportunity recognition: effects of mentors, industry networks, and professional forums. J. Bus. Ventur. 22, 174–192. 10.1016/j.jbusvent.2005.12.001

[B71] Pandit D. Joshi M. P. Tiwari S. R. (2018). Examining entrepreneurial intention in higher education: an exploratory study of college students in India. J. Entrep. 27, 25–46. 10.1177/0971355717738595

[B72] Patzelt H. Shepherd D. A. (2010). Recognizing opportunities for sustainable development. Entrep. Theory Pract. 35, 631–652. 10.1111/j.1540-6520.2010.00386.x

[B73] Pavlov G. Maydeu-Olivares A. Shi D. (2021). Using the standardized root mean squared residual (SRMR) to assess exact fit in structural equation models. Educ. Psychol. Meas. 81, 110–130. 10.1177/0013164420926231 33456064 PMC7797960

[B74] Podsakoff P. M. MacKenzie S. B. Lee J.-Y. Podsakoff N. P. (2003). Common method biases in behavioral research: a critical review of the literature and recommended remedies. J. Appl. Psychol. 88, 879–903. 10.1037/0021-9010.88.5.879 14516251

[B75] Podsakoff P. M. Organ D. W. (1986). Self-reports in organizational research: problems and prospects. J. Manage. 12, 531–544. 10.1177/0149206386012004088452065

[B76] Preacher K. J. Hayes A. F. (2008). Asymptotic and resampling strategies for assessing and comparing indirect effects in multiple mediator models. Behav. Res. Methods 40, 879–891. 10.3758/BRM.40.3.879 18697684

[B77] Sakib M. N. Rabbani M. R. Hawaldar I. T. Jabber M. A. Hossain J. Sahabuddin M. (2022). Entrepreneurial competencies and SMEs' performance in a developing economy. Sustainability 14, 13643. 10.3390/su142013643

[B78] Saleem M. S. Isha A. S. N. Yusop Y. M. Awan M. I. Naji G. M. A. (2022). The role of psychological capital and work engagement in enhancing construction workers' safety behavior. Front. Public Health 10, 810145. 10.3389/fpubh.2022.810145 35317512 PMC8934392

[B79] Schlaegel C. Koenig M. (2013). Determinants of entrepreneurial intent: a meta-analytic test and integration of competing models. Entrepren. Theory Pract. 38, 291–332. 10.1111/etap.12087

[B80] Shane S. Venkataraman S. (2000). The promise of entrepreneurship as a field of research. Acad. Manag. Rev. 25, 217–226. 10.5465/amr.2000.2791611

[B81] Shapero A. Sokol L. (1982). “The social dimensions of entrepreneurship,” in Kent C, Sexton D, Vesper KH, editors. The Encyclopedia of Entrepreneurship (Englewood Cliffs, NJ: Prentice-Hall).

[B82] Shepherd D. A. Williams T. A. Patzelt H. (2015). Thinking about entrepreneurial decision making: review and research agenda. J. Manag. 41, 11–46. 10.1177/0149206314541153

[B83] Sheu H.-B. Lent R. W. Brown S. D. Miller M. J. Hennessy K. D. Duffy R. D. (2010). Testing the choice model of social cognitive career theory across Holland themes: a meta-analytic path analysis. J. Vocat. Behav. 76, 252–264. 10.1016/j.jvb.2009.10.015

[B84] Shi Y. Yuan T. Bell R. Wang J. (2020). Investigating the relationship between creativity and entrepreneurial intention: the moderating role of creativity in the theory of planned behavior. Front. Psychol. 11, 1209. 10.3389/fpsyg.2020.01209 32581972 PMC7296068

[B85] Shu R. Ren S. Zheng Y. (2018). Building networks into discovery: the link between entrepreneur network capability and entrepreneurial opportunity discovery. J. Bus. Res. 85, 197–208. 10.1016/j.jbusres.2017.12.048

[B86] Song G. Min S. Lee S. Seo Y. (2017). The effects of network reliance on opportunity recognition: a moderated mediation model of knowledge acquisition and entrepreneurial orientation. Technol. Forecast. Soc. Chang. 117, 98–107. 10.1016/j.techfore.2017.01.004

[B87] Soomro B. A. Shah N. (2022). Entrepreneurship education, entrepreneurial self-efficacy, need for achievement and entrepreneurial intention among commerce students in Pakistan. Educ. Train. 64, 107–125. 10.1108/ET-01-2021-0023

[B88] Su X. Liu S. Zhang S. Liu L. (2020). To be happy: a case study of entrepreneurial motivation and entrepreneurial process from the perspective of positive psychology. Sustainability 12, 584. 10.3390/su12020584

[B89] Tan L. P. Pham L. X. Bui T. T. (2021). Personality traits and social entrepreneurial intention: the mediating effect of perceived desirability and perceived feasibility. J. Entrep. 30, 56–80. 10.1177/0971355720974811

[B90] Wang L. Huang J. (2022). Mediating role of entrepreneurial self-efficacy and prosocial tendency in the relation between college students' post-traumatic growth and entrepreneurial intention in the post-COVID-19 era. Front. Psychol. 13, 861484. 10.3389/fpsyg.2022.861484 35465517 PMC9021958

[B91] Wang R. Zhou H. Wang L. (2022). The influence of psychological capital and social capital on the entrepreneurial performance of the new generation of entrepreneurs. Front. Psychol. 13, 832682. 10.3389/fpsyg.2022.832682 35615164 PMC9126072

[B92] Wang W. Tang Y. Liu Y. Zheng T. Liu J. Liu H. (2019). Can sense of opportunity identification efficacy play a mediating role? Relationship between network embeddedness and social entrepreneurial intention of university students. Front. Psychol. 10, 1342. 10.3389/fpsyg.2019.01342 31244736 PMC6579831

[B93] Wang Z. Ortiz G. G. R. (2022). Assessing the management student's entrepreneurial intentions: role of entrepreneurship education and technology transfer. Front. Psychol. 13, 953324. 10.3389/fpsyg.2022.953324 36003108 PMC9393513

[B94] Wolf E. J. Harrington K. M. Clark S. L. Miller M. W. (2013). Sample size requirements for structural equation models: an evaluation of power, bias, and solution propriety. Educ. Psychol. Meas. 73, 913–934. 10.1177/0013164413495237 25705052 PMC4334479

[B95] Wu W. Wang H. Zheng C. Wu Y. J. (2019). Effect of narcissism, psychopathy, and machiavellianism on entrepreneurial intention-the mediating of entrepreneurial self-efficacy. Front. Psychol. 10, 360. 10.3389/fpsyg.2019.00360 30846958 PMC6393355

[B96] Xie S. Luo J. Zheng Y. Ma C. (2022). Entrepreneurship education of college students and entrepreneurial psychology of new entrepreneurs under causal attribution theory. Front. Psychol. 13, 943779. 10.3389/fpsyg.2022.943779 36405168 PMC9669758

[B97] Yang Q. Al Mamun A. Jingzu G. Siyu L. Masud M. M. (2023). Social entrepreneurial intention among working adults: an emerging country context. Front. Psychol. 14, 1123198. 10.3389/fpsyg.2023.1123198 36860787 PMC9968742

[B98] Zahra S. A. Gedajlovic E. Neubaum D. O. Shulman J. M. (2009). A typology of social entrepreneurs: motives, search processes and ethical challenges. J. Bus. Ventur. 24, 519–532. 10.1016/j.jbusvent.2008.04.007

[B99] Zhang X. Sun Y. Gao Y. Dong Y. (2022). Paths out of poverty: social entrepreneurship and sustainable development. Front. Psychol. 13, 1062669. 10.3389/fpsyg.2022.1062669 36533010 PMC9748285

[B100] Zhao J. Wei G. Chen K.-H. Yien J.-M. (2020). Psychological capital and university students' entrepreneurial intention in china: mediation effect of entrepreneurial capitals. Front. Psychol. 10, 2984. 10.3389/fpsyg.2019.02984 32038375 PMC6989491

